# 

**Published:** 1888-09

**Authors:** 


					﻿Vol. VIII.
SEPTEMBER, 1888.
No. 9.
urns
HOMEOPATHIC PHYSICIAN,
A Monthly Journal of Homoeopathic Materia Medica
and Clinical Medicine.
“ He is a freeman whom truth makes free, and all are slqyes beside,”
“Seek the Truth: come whence it may, cost what it will.”
EDITED AND PUBLISHED BY
EDMUND J. LEE, M. IX,
AND
WALTER <M. JAMES. ML D.
PHILADELPHIA :
No. 1133 SPRUCE 8TREET.
T. O 2ST X> O TT t
ALFRED HEATH & CO., Sole Agents for Great Britain,
114 Ebury Street, Eaton Square.
49-Yearly Subscription, $2.SO, invariably in advance. Single numbers, 2S cts.
Entered at the Philadelphia Poet-Office M Second-Class Mall Matter.
				

